# Relationship between Organizational Culture, Leadership Behavior and Job Satisfaction

**DOI:** 10.1186/1472-6963-11-98

**Published:** 2011-05-14

**Authors:** Yafang Tsai

**Affiliations:** 1 Department of Health Policy and Management, Chung Shan Medical University; Taiwan; 2 Department of Medical Management, Chung Shan Medical University Hospital; Taiwan

## Abstract

**Background:**

Organizational culture refers to the beliefs and values that have existed in an organization for a long time, and to the beliefs of the staff and the foreseen value of their work that will influence their attitudes and behavior. Administrators usually adjust their leadership behavior to accomplish the mission of the organization, and this could influence the employees' job satisfaction. It is therefore essential to understand the relationship between organizational culture, leadership behavior and job satisfaction of employees.

**Methods:**

A cross-sectional study was undertaken that focused on hospital nurses in Taiwan. Data was collected using a structured questionnaire; 300 questionnaires were distributed and 200 valid questionnaires were returned. To test the reliability of the data, they were analyzed by Cronbach's α and confirmatory factors. Correlation analysis was used on the relationships between organizational cultures, leadership behavior and job satisfaction.

**Results:**

Organizational cultures were significantly (positively) correlated with leadership behavior and job satisfaction, and leadership behavior was significantly (positively) correlated with job satisfaction.

**Conclusions:**

The culture within an organization is very important, playing a large role in whether it is a happy and healthy environment in which to work. In communicating and promoting the organizational ethos to employees, their acknowledgement and acceptance of it can influence their work behavior and attitudes. When the interaction between the leadership and employees is good, the latter will make a greater contribution to team communication and collaboration, and will also be encouraged to accomplish the mission and objectives assigned by the organization, thereby enhancing job satisfaction.

## Background

Organizational culture is described by Robbins & Coulter [1] as the shared values, beliefs, or perceptions held by employees within an organization or organizational unit. Because organizational culture reflects the values, beliefs and behavioral norms that are used by employees in an organization to give meaning to the situations that they encounter, it can influence the attitudes and behavior of the staff [2]. Understanding the organization's core values can prevent possible internal conflict [3], which is the main reason for our research into these cultural issues.

In other management fields, empirical research of organizational culture has involved the functionalist perspective, providing impressive evidence of the role of organizational culture in improving performance [4]. The pervasiveness of an organizational culture requires that management recognize its underpinning dimensions and its impact on employee-related variables, such as job satisfaction [5], organizational commitment [6], and performance [7]. Lund [5] believed that less research was done on the relationship between organizational culture and job satisfaction within the research topic of organizational culture and outcome. The organization consists of the staff, with the behavior of its individual members affecting outcomes. Since cultural research within the nursing field is not common [8], it is necessary to explore the way the culture influences the behavior of the nursing staff, and in turn how the behavior of the staff influences the organizational outcome.

A two-dimensional model of leadership that focuses on the concern for people and production has been used for many years in organizational research [9]. In the late 1970s, leadership research started focusing on behavior within organizational change and development [10]. Leadership implies authority in the broadest sense of the word and not simply the power to wield the stick [11]. It is based on objective factors, such as managerial ability, and more subjective characteristics that include personal qualities of the leaders. The factors are of even greater importance given the current emerging culture of the nurse who has a clear and assertive vision about the nature of clinical practice [12].

Currently, there is a shortage of nurses in clinical care, and good leaders can help any attrition. Furthermore, the leadership skills of nurse administrators can contribute to the success of their organization [13]. Leadership is of increasing importance in clinical nursing [14]. Although leadership and organizational culture constructs have been well studied, the relationship between them has not been established in the field of nursing [6]. This study explores the relationship between organizational culture and leadership behavior.

Berson & Linton [15] discovered that within the research & development (R&D) and administrative environments, leadership behavior of a manager is closely related to work satisfaction of the employees. Nielsen et al. [16] have stated that leadership behavior and job satisfaction will depend on the organizational context; therefore another objective of this research was to understand how the leadership behavior of the administrator in different organizational cultures affects job satisfaction. Casida & Pinto-Zipp [17] explored how nurses felt about the relationship between leadership and organizational culture, and found them to be correlated. Although the data indicated that the development of an organizational culture is related to the behavior of its leaders, the results failed conclude whether this affected their attitudes or behavior as employees. From the nursing administration perspective, the normal course of action taken to influence employee behavior and achieve the objectives set by the administrators comes through administrative management. Therefore, as well as discussing the relationship between leadership behavior and organizational culture, this research will investigate the effect of leader behavior and organizational culture towards employee job satisfaction. The findings clearly show that hospital administrators should be concerned about the effects of leadership behavior and organizational culture on the attitude towards work of their employees. This should help administrators alter their behavior in order to maintain a good mutual relationship with their subordinates, improving their working attitude and, more importantly, reducing potential conflicts.

### Relationship between organizational culture and leadership behavior

Culture is socially learned and transmitted by members; it provides the rules for behavior within organizations [18]. The definition of organizational culture is of the belief that can guide staff in knowing what to do and what not to do, including practices, values, and assumptions about their work [19]. The core values of an organization begin with its leadership, which will then evolve to a leadership style. Subordinates will be led by these values and the behavior of leaders, such that the behavior of both parties should become increasingly in line. When strong unified behavior, values and beliefs have been developed, a strong organizational culture emerges. Leaders have to appreciate their function in maintaining an organization's culture. This would in return ensure consistent behavior between members of the organization, reducing conflicts and creating a healthy working environment for employees [20].

*Hypothesis 1- Organizational culture is positively correlated with leadership behavior*.

### Relationship between leadership behavior and job satisfaction

Job satisfaction has been associated with nurses who perceive their managers as supportive and caring. A supportive manager shares values, believes in a balance of power, and provides opportunities for open dialogue with nurses [21], which in turn reduces the chances of internal conflicts. This type of leader is successful in his or her role and is supportive and responsive to clinical nurses, thereby preserving power and status within the hospital system. Such leaders are valued throughout the organization and have executive power to do what they see as necessary to create a positive environment for nursing [22]. Accordingly, they have a measurable effect on the morale and job satisfaction of nurses [23].

*Hypothesis 2 - Leadership behavior is positively correlated with job satisfaction*.

### Relationship between organizational culture and job satisfaction

Organizational culture expresses shared assumptions, values and beliefs, and is the social glue holding an organization together [24]. A strong culture is a system of rules that spells out how people should behave [25]. An organization with a strong culture has common values and codes of conduct for its employees, which should help them accomplish their missions and goals. Work recognition and job satisfaction can be achieved when employees can complete the tasks assigned to them by the organization.

*Hypothesis 3 -.Organizational culture is positively correlated with job satisfaction*.

### The measurement of organizational culture, leadership behavior and job satisfaction

A structured questionnaire was compiled based on similar studies published in international journals [26,27]. Twenty-three factors regarding organizational culture were taken from Tsui et al. [26], a study based on two groups of MBA students from two universities in Beijing, China. Our research was focused on clinical nurses in hospitals; therefore, refinements were made to the questionnaire designed by Tsui et al. [26] to cater for our particular research objective. The study invited three directors or supervisors from the medical center to validate the questionnaire. Lastly, there were 22 questions in the organizational culture section.

Thirty items regarding leadership behavior were taken from Strange & Mumford [27], and the questions structured using this literature. However, the proposed test was not empirically studied. Nurses from hospital A were used as a pilot study sample. Four question items were deleted to improve the validity of the questionnaire: "People will have an extreme reaction to the leader"; "Followers will sacrifice themselves for the leader and/or the leader's vision"; "The leader is motivated by the accomplishment of his vision"; and "The leader will take into account the needs of the organization in his decision making."

Vroom [28] classified job satisfaction into 7 dimensions: organizational, promotion, job content, superior, reward, working environment and working partners. We took into consideration that nurses' salary increases are based on promotion. Furthermore, a large number of variables in organization culture and leadership behavior were covered by this research. To prevent too few number nurses from responding to the questionnaires, we asked only 4 job satisfaction dimensions out of a total of 12 items: job recognition, reward and welfare, superior and working partners.

## Methods

### Study Design

A cross-sectional study was conducted in two hospitals in Central Taiwan.

### Data Source and Analysis

We employed self-administered questionnaires to collect research data. Data was collected between October 1 and November 30, 2008. We selected 2 hospitals as our sample target and appointed a designated person at each to issue questionnaires to employees. The number of questionnaires issued depended on the designated person. The questionnaires were completed voluntarily by all respondents. During the research period, there were 325 nurses in hospital A; 100 questionnaires were distributed, and 57 valid questionnaires were returned. In hospital B there were a total of 572 nurses; 200 questionnaires were distributed, and 143 valid questionnaires were returned (total return rate 66.7%).

Of the subjects, 99.5% were female, 83.5% single or never married, 35.5% had a tenure at the hospital of 1-2 years, and 45.0% had had a college-level education. The majority of employees at the hospitals were general nurses (89.5%), and the average age was between 21 and 30 years (82.5%)(see Table 1).

**Table 1:** Participant Demographics

Variables	Number	%
Gender *(n = 200)*		
Female	199	99.5
Male	1*	0.5
Marital status *(n = 200)*		
Married	33	16.5
Single, never married	167	83.5
Tenure *(n = 200)*		
<1 years	42	21.0
1-2 years	71	35.5
3-4 years	36	18.0
5-6 years	13	6.5
7-9 years	27	13.5
>= 10 years	11	5.5
Educational level *(n = 200)*		
College	90	45.0
University	62	31.0
Postgraduate	48	24.0
Position in hospital *(n = 200)*		
General Nurse	179	89.5
Head Nurse	21	10.5
Age *(years, n = 200)*		
21-30	165	82.5
31-40	33	16.5
41-50	2	1.0

Note:* The gender of the majority nurses in Taiwan are female. Within the research sample there is only 1 male nurse.

All data were analyzed using the SPSS 17.0 software package. Cronbach's α coefficient was used to assessed the internal consistency reliability of scales. To explore the factor construct of scale, a series of exploratory factor analysis (EFA) were employed. Correlation analysis was used to test for the relationships among subscales of organizational culture, leadership behavior and job satisfaction scale. Finally, a series of regression analysis were used to identify the proposed hypotheses. For H1 and H3, two sets of simple linear regression were used to assess the association between independent variable and dependent variable. For H2, hierarchical regression analysis was used to assess the independent association between leadership behavior and job satisfaction after controlling for the effect of organizational culture. Partial *R*^2 ^(Δ*R*^2^), *F *test and standardized regression coefficient (*β*) and their test statistics (*t *value) were reported in all regression analysis.

### Measurement

Given the latent character of the variables considered in the study, we used multi-item, 5-point Likert-type scales (1='strongly disagree' and 5='strongly agree'). After reliability analysis, the Cronbach's α of the organizational culture scale was 0.958 (22 items). The Cronbach's α of the leadership behavior scale was 0.966 (26 items), and for job satisfaction 0.855 (12 items).

The questionnaires used exploratory factor analysis. We extracted 4 factors from the organizational culture via principal component analysis, used the Varimax of the rotation method, and named them: employee orientation, customer focus, emphasizing responsibility, and emphasizing cooperation. We extracted 4 factors from leadership behavior and named them: leader's encouragement and supportiveness to subordinates, leader giving subordinates a clear vision and trust, leader's behavior is consistent with organization's vision, and leader is persuasive in convincing subordinates to acknowledge the vision. We extracted factors for job satisfaction and called them: working partners, rewards and welfare, superior and job recognition.

## Results

### Descriptive statistics

The average score for organizational culture was between 3.73 and 3.19, but the highest score was 3.73: "satisfying the need of customers at the largest scale." The second highest score was 3.68: "the profit of the customer is emphasized extremely." The lowest score was 3.19: "concern for the individual development of employees" (see Table 2).

**Table 2:** Mean and Standard Division and the Factor Analysis of Organizational Culture, Leadership Behavior and Job Satisfaction

Dimensions	Items	Mean	Standard Division	Factor Loading	Rotation Sums of squared loadings total	Percentage of variance explained (%)	Percentage of cumulative variance explained (%)
Employee orientation (OC1)	■ Concerning for the individual development of employees.	3.19	0.926	0.748	4.543	20.651	20.651
	■ Caring about opinions from employees.	3.18	0.960	0.778			
	■ Adopting high-tech bravely.	3.20	0.895	0.604			
	■ Having a clear standard on praise and punishment.	3.24	0.985	0.776			
	■ Possessing a comprehensive system and regulations.	3.21	0.885	0.806			
	■ Setting clear goals for employees.	3.23	0.891	0.766			

Customer focus (OC2)	■ Sincere customer service.	3.50	0.908	0.693	4.292	19.509	40.159
	■ Customer is number one.	3.60	0.962	0.781			
	■ Providing first class service to customers.	3.62	0.943	0.740			
	■ The profit of the customer is emphasized extremely.	3.68	0.855	0.785			
	■ Developing new products and services continuously.	3.61	0.873	0.779			
	■ Ready to accept new changes.	3.50	0.874	0.606			

Emphasizing responsibility (OC3)	■ Consideration among employees.	3.66	0.785	0.644	4.100	18.638	58.797
	■ Satisfying the need of customers at the largest scale.	3.73	0.806	0.678			
	■ Emphasizing innovation.	3.42	0.810	0.670			
	■ Keeping strictly working disciplines.	3.63	0.822	0.750			
	■ Showing social responsibility.	3.58	0.858	0.631			
	■ Emphasizing on economic and social profits.	3.49	0.857	0.618			

Emphasizing cooperation (OC4)	■ Consideration among employees.	3.38	0.849	0.850	3.534	16.065	74.862
	■ Satisfying the need of customers at the largest scale.	3.59	0.840	0.797			
	■ Emphasizing innovation.	3.62	0.830	0.789			
	■ Keeping strictly working disciplines.	3.54	0.838	0.667			

Leader's encouragement and supportive to subordinates (LB1)	■ The leader will express high performance expectations for followers.	3.58	0.772	0.671	0.6959	26.766	26.766
	■ The leader will communicate a high degree of confidence in the followers' ability to meet expectations.	3.64	0.827	0.678			
	■ The leader will demonstrate behaviors that selectively arouse unconscious achievement, power, and affinitive motives of followers when these motives are specifically relevant to the attainment of the vision.	3.61	0.862	0.767			
	■ Leadership occurs through articulation of the vision and accomplishments that pertain to vision attainment.	3.61	0.923	0.757			
	■ Followers are attracted to the leader himself.	3.64	0.886	0.692			
	■ For the leader to be effective there must be some catalyst to make the followers open to the leader and her/his vision.	3.43	0.818	0.725			
	■ The leader will allow followers the autonomy to make their own decisions but will influence them to make decisions in line with her/his vision.	3.60	0.862	0.705			
	■ The leader will back up orders with justification based on the goodness of her/his vision.	3.62	0.831	0.710			
	■ Followers are directly influenced by the leader and their personal relationship with her/him.	3.44	0.836	0.667			
	■ The leader cares about his image and plays to the desires of followers.	3.53	0.902	0.674			
	■ The leader will take an interest in all current and potential followers.	3.65	0.808	0.499			
	■ Followers are devoted and unquestioning of the leader.	3.54	0.749	0.566			

Leader giving subordinate her/his clear vision (LB2)	■ The leader will negotiate her/his ideas when it benefits her/his image or her/his vision.	3.66	0.822	0.514	4.705	18.098	44.863
	■ The leader will use positive rewards and reinforcement with her/his followers.	3.69	0.804	0.524			
	■ The leader may change her/his vision to meet the needs and wants of the followers and the organization.	3.51	0.789	0.548			
	■ The leader will exude confidence, dominance, and a sense of purpose.	3.62	0.817	0.633			
	■ The leader will motivate the followers to act upon ideas already in place in society.	3.57	0.733	0.731			
	■ The leader will be narcissistic and wish to bring power and attention to herself/himself.	3.66	0.746	0.802			
	■ The leader will interact with followers-social distance is low.	3.67	0.862	0.762			
Leader's behavior is consistent with her/his vision (LB3)	■ The leader will act accordingly to certain vision that specifies a better future state.	3.77	0.855	0.734	4.317	16.606	61.469
	■ The leader will strive toward distal rather than proximal goals.	3.72	0.847	0.789			
	■ The leader will communicate messages that contain references to her/his overall vision.	3.68	0.843	0.784			
	■ The leader will behaviorally role model the values implied by the vision by personal example.	3.77	0.781	0.561			

Leader is persuasive in convincing subordinates to acknowledging her/his vision (LB4)	■ The leader will excel in persuading people to agree with her/him.	3.50	0.862	0.494	2.580	9.923	71.392
	■ The leader will try to persuade those who disagree with her/his vision to agree with it.	3.42	0.759	0.883			
	■ The leader will delegate authority for the attainment of her/his vision.	3.58	0.739	0.655			

Working partners (JS1)	■ I am satisfied with the communication status between colleagues within my department.	3.79	0.767	0.602	2.501	20.839	20.839
	■ I am satisfied with the communication status between my department and other departments.	3.48	0.736	0.809			
	■ I am satisfied with the team I worked with in my department as well as other departments.	3.56	0.748	0.880			
	■ I am satisfied with the team formed within my own department.	3.67	0.765	0.709			

Rewards and welfare (JS2)	■ I am satisfied with my remuneration because by comparing the amount of workload with other department, I actually have less workload.	2.56	1.198	0.925	2.397	19.973	40.812
	■ I am satisfied with the welfare provided by the hospital.	2.74	1.100	0.903			

Superior (JS3)	■ Whenever I require assistance, a supervisor is always there to help.	3.77	0.781	0.869	2.302	19.182	59.994
	■ A particular supervisor will always listen to my issues and assist me in resolving those issues.	3.66	0.780	0.770			
	■ Until now I am very satisfied with my job.	3.39	0.895	0.575			

Job recognition (JS4)	■ I will be recognized when I perform outstandingly.	3.61	0.749	0.722	1.969	16.405	76.400
	■ I will be rewarded if I provided good service to the patients.	3.41	0.863	0.745			
	■ My role is considered very important to some people.	3.84	0.786	0.611			

Note: The leader is mean nursing director.

The average score for leadership behavior was between 3.77 and 3.42, where 2 items scored the highest score at 3.77: "the leader will act accordingly with a certain 'vision' that specifies a better future state", and "the leader will behaviorally role model the values implied by the vision by personal example". The second highest score was 3.69: "the leader will use positive rewards and reinforcement with his followers." The lowest score was 3.42: "the leader will try to persuade those who disagree with his vision to agree with it" (see Table 2).

The average score for job satisfaction was between 3.84 and 2.56, where the highest score was 3.84: "to certain people my work is extremely important." The second highest score was "I am satisfied with how colleagues communicate with each other in the office." The lowest score was 2.56: "I am satisfied with my salary as I have less workload compared to other employees in other divisions" (see Table 2).

### Inferential statistical analysis

In relation to the 4 dimensions of organizational culture (employee orientation, customer focus, emphasizing responsibility, and emphasizing cooperation), the 4 dimensions of leadership behavior (leader's encouragement and support to subordinates, leader giving subordinates her/his clear vision, leader's behavior is consistent with the her/his vision and leader is persuasive in convincing subordinates to acknowledge the her/his vision), and the 4 dimensions of job satisfaction (working partners, rewards and welfare, superior and job recognition), variable analysis was carried out. The results of the analysis showed that only 2 dimensions from "leader giving subordinates her/his clear vision" and "behavior consistent with her/his vision" and "reward and welfare" under the job satisfaction were not significantly correlated, whereas the other dimensions showed significant correlation. The results also showed that organizational culture, leadership behavior and job satisfaction were positively associated with hypotheses one to three, which were supported (see Table 3).

**Table 3:** Correlation Analysis among Organizational Culture, Leadership Behavior and Job Satisfaction

Variables	Organizational Culture				Leadership Behavior				Job Satisfaction			
Dimensions	OC1	OC2	OC3	OC4	LB1	LB2	LB3	LB4	JS1	JS 2	JS 3	JS 4
OC1	1											
OC2	0.604**	1										
OC3	0.597**	0.782**	1									
OC4	0.678**	0.618**	0.601**	1								
LB1	0.512**	0.359**	0.366**	0.405**	1							
LB2	0.413**	0.489**	0.552**	0.523**	0.770**	1						
LB3	0.345**	0.449**	0.444**	0.453**	0.746**	0.744**	1					
LB4	0.297**	0.338**	0.406**	0.343**	0.562**	0.632**	0.516**	1				
JS1	0.350**	0.360**	0.390**	0.381**	0.439**	0.411**	0.329**	0.341**	1			
JS2	0.521**	0.234**	0.145*	0.352**	0.174*	0.138	0.057	0.165*	0.170*	1		
JS3	0.481**	0.386**	0.427**	0.407**	0.657**	0.539**	0.447**	0.313**	0.502**	0.338**	1	
JS4	0.530**	0.357**	0.341**	0.374**	0.906**	0.698**	0.607**	0.503**	0.362**	0.234**	0.575**	1

Note **Correlation is significant at the 0.01 level (2-tailed). * Correlation is significant at the 0.05 level (2-tailed).

**Figure 1:**
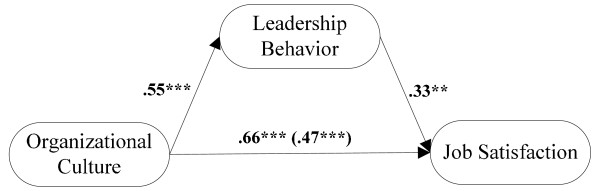
**The association between organizational culture, leadership behavior and job satisfaction**. (The values shown were standardized regression coefficient and value in parenthesis was partially standardized regression coefficient)

Table 4 presents the results of several regression analyses. H1 was supported, as organizational culture was positively associated with leadership behavior (*β *= .55, *p *< .001). H3 was also supported as organizational culture was positively related to job satisfaction (*β *= .66, *p *< .001). Finally, H2 was supported as the partial regression coefficient of leadership behavior reached statistically significant (*β *= .33, *p *< .001) after controlling the effect of organizational culture. The unique variance explained attributable to leadership behavior was 8% (Δ*F *= 30.58, *p*<.001) independent of organizational culture (see Table 4). The association among there three main variables was illustrated as Figure 1.

**Table 4:** The Linear Regression of Organizational Culture, Leadership Behavior and Job Satisfaction

	Leadership Behavior (H1)	Job Satisfaction (H3)	Job Satisfaction (H2)
		
	*β *(*t *value)	*β *(*t *value)	*β *(*t *value)
Organizational Culture	.55 (9.37***)	.66 (12.26***)	.47 (7.87***)
Leadership Behavior	--	--	.33 (5.53***)
Δ*R*^2^	.31	.43	.08†
Δ*F *value	87.83***	150.29***	30.58***
Overall *R*^2^	.31	.43	.51
Overall *F *value	87.83***	150.29***	101.66***
Degree of freedom	1, 198	1, 198	2, 197

*Note*: *** *p *<.001, *β*= standardized regression coefficient, *t *value = test statistics of *β*,

†Δ *R*^2^= is the unique variance attributable to leadership behavior independent of organizational culture in the hierarchical regression analysis.

## Discussion

Casida & Pinto-Zipp [17] studied nurses in determining the relationship between different leadership styles and organizational cultures, and showed a correlation between leadership and organizational culture, consistent with the findings of our research. However, by adopting regression analysis, we also found that leadership behavior impacts on organizational culture.

Laschinger et al. [29] proposed that the variables strongly correlated with job satisfaction included role conflict, head nurse leadership, supervisory relationships, autonomy, and stress. Mayo [30] argued that the key determinant of job satisfaction was group interaction, and highlighted the importance of good leadership and satisfying personal relations in the workplace. Management and leadership behavior at the hospital affected nurses' job satisfaction [31]. The research also discovered that leadership behavior will also influence employee job satisfaction. As well as the above-described individual factors, the research also showed that factors at the organization level, such as the organizational culture, also have an effect on job satisfaction. This result is consistent with the results of Gifford et al. [32]. It is recommended that it is also important for hospital administrators to establish a good organizational infrastructure in addition to improving the working environment in order to increase employee job satisfaction.

Decisions about patient care are often made by a team, rather than by a single individual [33]. To maintain open communication and better coordination, as well as avoiding possible conflicts, one must rely on the role of leaders to motivate the team to achieve the organization goal. It was found that encouragement and support by leaders, their trust and clear vision, their consistent behavior in this regard and their ability to convince subordinates to acknowledge their vision, can all influence employee job satisfaction. On the other hand, we found that the factors in achieving job satisfaction were not limited to the employee's working environment, but also included interactions between working partners. Good health care requires good team behavior, so it is also recommended that hospital administrators not only establish relationships within the health care teams, but also work to improve these relationships to increase employee job satisfaction.

Academics who study organizational culture as their research topic feel that organizational culture is complex. It will influence different employee attitudes and behavior [34]; for example Jacobs & Roodt [35] discovered a correlation between employee turnover intentions, knowledge sharing organizational commitment, organizational citizenship behavior, job satisfaction and organizational culture. Other academics have found that organizational culture is also related to organization or employee efficiency. Good examples are an organization's innovative ability [36], employee effectiveness (e.g. higher levels of goal orientation, self control) [37]. Kane-Urrabazo [20] believed that a satisfactory work environment can be created by the employees when an organisation possesses a healthy culture and thus has a positive attitude towards employee work. Therefore the relationship between organisational culture and employee behaviour/attitude has been emphasised by different academics from various fields [26]. Jacobs & Roodt [35] showed a positive correlation between organisational culture and employee job satisfaction that is consistent with the findings of our research.

### Research limitations and future research

Since a wide range of variables were included in our study, only a limited number of clinical nurses were interested in participating. Furthermore, only 2 hospitals were involved in this research; therefore, it is proposed that in view of the response rate, future research should consider adjusting the research variables.

Organizations face challenges in the external environment and changing internal context, and leaders will alter their behavior to adapt to these environment changes. Therefore it is proposed that longitudinal research methods can be adopted in future investigations into how changes in organizational context impact on leadership behavior. Will these changes create a brand new organization culture? And how will these changes in leadership behavior influence employee behavior and their contribution to the organization?

Administrators usually adjust their leadership behavior in order to reach the organizational goal. It is proposed that future research can explore the type of leadership behavior that will shape a particular culture within an organization. Thus, administrators can achieve the objective of shaping a new organization culture by adopting different leadership behavior training programs.

## Conclusion

Culture within an organization is very important, playing a large role in whether or not the organization is a happy and healthy place to work [20]. Through communicating and promoting the organizational vision to subordinates, and in getting their acknowledgement of the vision, it is possible to influence their work behavior and attitudes. When there is good interaction between the leader and subordinates, there will be contributions to team communication and collaboration, and encouragement of subordinates to accomplish the mission and objectives assigned by the organization, which in turn enhances job satisfaction.

## Pre-publication history

The pre-publication history for this paper can be accessed here:

http://www.biomedcentral.com/1472-6963/11/98/prepub

## References

[B1] Robbins Stephen P Coulter M Management 2005 8 Pearson Prentice Hall

[B2] Scott-Findlay Shannon Estabrooks Carole A Mapping the organizational culture research in nursing. In: A literature review Journal of Advanced Nursing 2006 56 5 498 513 10.1111/j.1365-2648.2006.04044.x 17078826

[B3] Watson Bill Clarke Charlotte Swallow Vera Forster Stewart Exploratory factor analysis of the research and development culture index among qualified nurses Journal of Clinical Nursing 2005 14 1042 1047 10.1111/j.1365-2702.2005.01214.x 16164521

[B4] Denison DR Mishra AK Toward a theory of organizational culture and effectiveness Organization Science 1995 6 204 223 10.1287/orsc.6.2.204

[B5] Lund Daulatram B Organizational culture and job satisfaction Journal of Business & Industrial Marketing 2003 18 3 219 236 10.1108/088586203104731312530436

[B6] Casida Jesus Pinto-Zipp Genevieve Leadership-organizational culture relationship in nursing units of acute care hospitals Nursing Economic 2008 26 1 7 15 18389837

[B7] Denison DR Haaland S Goelzer P Corporate culture and organizational effectiveness: Is Asia different form the rest of the world? Organizational Dynamics 2004 33 1 98 109 10.1016/j.orgdyn.2003.11.008

[B8] Cooke A Green B Developing the research capacity of departments of nursing and midwifery based in higher education: A review of the literature Journal of Advanced Nursing 2000 32 57 65 10.1046/j.1365-2648.2000.01447.x 10886435

[B9] Blake RR Mouton JS Theory and research for developing a science of leadership Journal of Applied Behavioural Science 1982 18 3 275 291 10.1177/002188638201800304

[B10] Anders Skogstad Einarsen Stasle The importance of a change-centred leadershipstyle in four organizational cultures Scandinavian Journal of Management 1999 15 289 306 10.1016/S0956-5221(98)00028-1

[B11] Lorentzon M Authority, leadership and management in nursing Journal of Advanced Nursing 1992 17 525 527 10.1111/j.1365-2648.1992.tb02826.x 1602065

[B12] Brendan McCormack Hopkins Eileen The development of clinical leadership through supported reflective practice Journal of Clinical Nursing 1995 4 161 168 7773525 10.1111/j.1365-2702.1995.tb00201.x

[B13] Perra Barbara Murdoch Leadership: The key to quality outcomes Journal of Nursing Care Quality 2001 15 2 68 73 10.1097/00001786-200115020-00008

[B14] Sandra Swearingen A journey to leadership: Designing a nursing leadership development program Journal of Continuing Education in Nursing 2009 40 3 107 112 10.3928/00220124-20090301-02 19326817

[B15] Berson Y Linton J An examination of the relationships between leadership behavior, and employee satisfaction in R & D versus administrative environments R & D Management 2005 35 51 6021584907

[B16] Karina Nielsen Yarker Joanna Brenner Sten-Olof Randall Raymond Borg Vilhelm The importance of transformational leadership style for the well-being of employees working with older people Journal of Advanced Nursing 2008 63 5 465 475 10.1111/j.1365-2648.2008.04701.x 18727749

[B17] Jesus Casida Pinto-Zipp Genevieve Leadership-organizational culture relationship in Nursing units of acute care hospitals Nursing Economic 2008 26 1 7 15 18389837

[B18] Jen-Te Yang Knowledge sharing: Investigating appropriate leadership roles and collaborative culture Tourism Management 2007 28 530 543 10.1016/j.tourman.2006.08.006

[B19] Staniland M What Is Political Economy? A Study of Social Theory and Underdevelopment 1985 Yale University Press

[B20] Christine Kane-Urrabazo Management's role in shaping organizational culture Journal of Nursing Management 2006 14 188 194 10.1111/j.1365-2934.2006.00590.x 16600006

[B21] Wade GH Osgood B Avino K Bucher G Bucher L Foraker T French D Sirkowski C Influence of organizational characteristics and caring attributes of managers on nurses' job enjoyment Journal of Advanced Nursing 2008 64 4 344 53 10.1111/j.1365-2648.2008.04775.x 18990113

[B22] Sullivan-Havens D Aiken LH Shaping systems to promote desired outcomes: The magnet hospital Journal of Nursing Administration 1999 29 14 19 10029797 10.1097/00005110-199902000-00006

[B23] Upenieks Valda Nurse leaders' perceptions of what compromises successful leadership in today's acute inpatient environment Nursing Administration Quarterly 2003 27 2 140 152 12765106 10.1097/00006216-200304000-00008

[B24] Trevino LK Nelson KA Managing business ethics: Straight talk about how to do it right 1999 2 John Wiley & Sons, Inc., New York, NY

[B25] Deal TE Kennedy AA Corporate cultures: The rites and rituals of corporate life 2000 Perseus Publishing, Cambridge, MA, USA

[B26] Tsui Anne S Zhang Zhi-Xue Wang Hui Xin Katherine R Wu Joshua B Unpacking the relationship between CEO leadership behavior and organizational culture Leadership Quarterly 2006 17 113 137 10.1016/j.leaqua.2005.12.001

[B27] Strange Jill M Mumford Michael D The origins of vision charismatic versus ideological leadership Leadership Quarterly 2002 13 343 377 10.1016/S1048-9843(02)00125-X

[B28] Vroom VH Ego involvement, job satisfaction, and job performance Personnel Psychology 1962 15 159 177 10.1111/j.1744-6570.1962.tb01858.x

[B29] Laschinger Heather K Spence Shamian Judith Thomson Donna Impact of magnet hospital characteristics on nurses' perceptions of trust, burnout, quality of care, and work satisfaction Nursing Economic 2001 19 5 209 219

[B30] Mayo E The social problems of an industrial civilization 1945 Boston: Division of Research, Graduate School of Business Administration, Harvard University

[B31] Elizabeth J Ann E The changing nature of nurses' job satisfaction: an exploration of sources of satisfaction in the 1990s Journal of Advanced Nursing 1999 30 1 150 158 10.1046/j.1365-2648.1999.01059.x 10403991

[B32] Gifford Blair D Zammuto Raymond F Goodman Eric A The relationship between hospital unit culture and nurses' quality of work life Journal of Healthcare Management 2002 47 1 13 25 11836962

[B33] Nancarrow Susan The impact of intermediate care services on job satisfaction, skills and career development opportunities Journal of Clinical Nursing 2007 16 1222 1229 10.1111/j.1365-2702.2007.01355.x 17584339

[B34] Van Der Post WZ De Coning TJ Smit EV An instrument to measure organizational culture South African Journal of Business Management 1997 28 4 147 168

[B35] Jacobs E Roodt G Organizational culture of hospitals to predict turnover intentions of professional nurses Health SA Gesondheid 2008 13 1 63 78

[B36] Funk SG Champagne MT Wises RA Tornquist EM BARRIERS-the barriers to research utilization scale Applied Nursing Research 1991 4 39 45 10.1016/S0897-1897(05)80052-7 1741634

[B37] Schneider WE Productivity improvement through cultural focus Consulting Psychology Journal: Practice and Research 1995 47 1 3 27

